# Influence of leaf vein density and thickness on hydraulic conductance and photosynthesis in rice (*Oryza sativa* L.) during water stress

**DOI:** 10.1038/srep36894

**Published:** 2016-11-16

**Authors:** Muhammad Adnan Tabassum, Guanglong Zhu, Abdul Hafeez, Muhammad Atif Wahid, Muhammad Shaban, Yong Li

**Affiliations:** 1 Ministry of Agriculture Key Laboratory of Crop Ecophysiology and Farming System in the Middle Reaches of the Yangtze River, College of Plant Science and Technology, Huazhong Agricultural University, Wuhan, Hubei, China; 2 Cotton Physiology Lab for Efficient Production, College of Plant Science and Technology, Huazhong Agricultural University, Wuhan, Hubei, China; 3 National Key Laboratory of Crop Genetic Improvement, College of Plant Science and Technology, Huazhong Agricultural University, Wuhan, Hubei, China

## Abstract

The leaf venation architecture is an ideal, highly structured and efficient irrigation system in plant leaves. Leaf vein density (LVD) and vein thickness are the two major properties of this system. Leaf laminae carry out photosynthesis to harvest the maximum biological yield. It is still unknown whether the LVD and/or leaf vein thickness determines the plant hydraulic conductance (*K*_plant_) and leaf photosynthetic rate (*A*). To investigate this topic, the current study was conducted with two varieties under three PEG-induced water deficit stress (PEG-IWDS) levels. The results showed that PEG-IWDS significantly decreased *A*, stomatal conductance (g_s_), and *K*_plant_ in both cultivars, though the IR-64 strain showed more severe decreases than the Hanyou-3 strain. PEG-IWDS significantly decreased the major vein thickness, while it had no significant effect on LVD. *A*, g_s_ and *K*_plant_ were positively correlated with each other, and they were negatively correlated with LVD. *A*, g_s_ and *K*_plant_ were positively correlated with the inter-vein distance and major vein thickness. Therefore, the decreased photosynthesis and hydraulic conductance in rice plants under water deficit conditions are related to the decrease in the major vein thickness.

Photosynthesis is an important physiological process that is very sensitive to abiotic stresses^1^,^2^. Diffusive (stomatal or mesophyll conductance) and biochemical impairments are considered two major responses that decrease photosynthesis under drought conditions^3^,^4^. Stomatal conductance (g_s_) is a fundamental process required for CO_2_ acquisition and is regulated by stomatal opening and closing^5^,^6^. A decreasing leaf turgor pressure and an increasing vapor pressure deficit (VPD) closes the stomata rapidly in response to water deficit condition^7^. Thus, stomatal limitation is a key cause of the decrease in *A* that occurs under water-limited conditions^7^,^8^.

The water transportation capacity of plants is known as the plant hydraulic conductance^9^,^10^, which is determined by the root, stem and leaf hydraulic conductance (*K*_leaf_)^11^. The root contribution ranges from one-third to one-half of the internal plant resistances^12^,^13^. The transpiration rate (*E*) or stomatal conductance exhibit significant and linear correlations with *K*_plant_ in a number of higher plants and rice plant^14^,^15^,^16^,^17^,^18^. Therefore, the capacity of water transport system controls the plant growth as it maintains the hydraulic link between the roots and leaves^19^.

The leaf hydraulic architecture is the key location for gas exchange between the plant and its environment^20^,^21^, and extra-vascular resistance imposes one-quarter or higher resistance (≥30%) in *K*_leaf_^22^,^23^. A decrease in *K*_leaf_ leads to stomatal closure, which reduces photosynthesis^24^,^25^. Therefore, a strong correlation has been observed between g_s_ and *K*_leaf_^22^,^23^,^26^. The leaf venation architecture is a perfect illustration of a highly efficient irrigation structure^27^,^28^. Veins are made up of phloem and xylem vessels implanted in parenchyma, rarely in sclerenchyma, that are wrapped in bundle sheath cells. Leaf veins in monocots of the *Poaceae* family are divided into three categories (major, minor and transverse veins) in addition to the leaf midrib, and are different in sizes and functions^29^,^30^,^31^,^32^,^33^. The major longitudinal veins run from the leaf lamina into the leaf sheath, while minor longitudinal veins mostly terminate at the junction of the leaf lamina and leaf sheath^34^,^35^,^36^,^37^.

The leaf venation architecture has many functions, including mechanical support^38^, sugars and hormone transportation^39^, and replacement of water lost through *E* during photosynthetic processes^23^. An enormous variation is found in the vein arrangement, size and density, and in the geometry of phloem and xylem vessels within the leaf vascular bundles. Thicker veins have a greater water transportation and sugar translocation capacity due to the greater number and/or size of the xylem and phloem vessels^40^.

During the last two decades, numerous studies have been carried out to explore the relationship between *K*_leaf_ and leaf vein structure. The leaf vein length per unit leaf area is called as the vein length per unit area (VLA) or leaf vein density (LVD). Positive, negative^41^,^42^ and no correlations^43^ have been found between LVD and *K*_leaf_ in these studies. In our previous study, a significant positive correlation between *K*_leaf_, *K*_plant_ and LVD was observed in rice plants under well watered condition, but no relationship was observed between *K*_leaf_ and *K*_plant_ under drought stress, although *K*_leaf_ showed a positive correlation with LVD^44^.

It is still unknown which vein property in rice crops is more closely related to the leaf photosynthetic rate and *K*_plant_ under drought conditions. The current study had the following objectives: (i) to elaborate the effects of PEG-induced water deficit stress (PEG-IWDS) on gas exchange parameters; (ii) to elaborate weather the LVD or leaf vein thickness is related to *K*_plant_; and (iii) to elaborate whether the LVD or leaf vein thickness is related to gas exchange parameters under PEG-IWDS.

## Results

**Figure 1:**
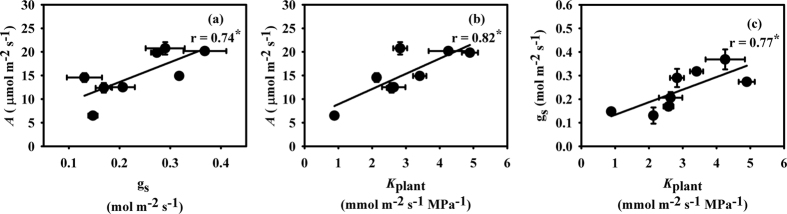
Relationships between photosynthesis (*A*) and stomatal conductance (g_s_) (**a**) and plant hydraulic conductance (*K*_plant_) (**b**) and relationship between g_s_ and *K*_plant_ (**c**). The data are presented as the mean values of 3 replicates. *P < 0.05.

PEG-induced water deficit stress decreased the gas exchange parameters. More severe depression was observed in the IR-64 variety than in the Hanyou-3 variety (Table 1). IR-64 had a significant decrease in *A* under all PEG-IWDS conditions, while *A* was decreased non significantly under 5% PEG-IWDS in Hanyou-3. Under 15% PEG-IWDS, *A* was decreased by 68.7% in IR-64 compared with a smaller decrease of 27.8% in Hanyou-3. Hanyou-3 showed a significant decrease in g_s_ under 15% PEG-IWDS, and IR-64 revealed a significant decrease in g_s_ under both the 10% and 15% PEG-IWDS conditions. The intercellular CO_2_ concentration (C_i_) increased under all stress levels in both varieties, but a significant increase was observed in IR-64 under 15% PEG-IWDS. Hanyou-3 and IR-64 both showed a significant decrease in *E* under 15% PEG-IWDS, but a more severe decrease (70.7%) was observed in IR-64 than Hanyou-3 (55.2%). The decrease in leaf water potential (Ψ_leaf_) was only significant in IR-64 under 15% PEG-IWDS. There was a positive relationship between *A* and g_s_ (Fig. 1a). *K*_plant_ showed a significant decrease in both varieties under 15% PEG-IWDS, although IR-64 showed a more severe decrease (68.8%) than Hanyou-3 (49.9%) (Table 1). *A* and g_s_ showed positive correlations with *K*_plant_ (Fig. 1b,c).

**Figure 2:**
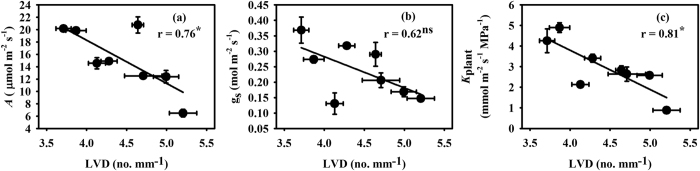
Relationship of photosynthesis (*A*) (**a**), stomatal conductance (g_s_) (**b**) and plant hydraulic conductance (*K*_plant_) (**c**) with leaf vein density (LVD). The data are presented as the mean values of 3 replicates. ns, not significant; *P < 0.05.

**Figure 3:**
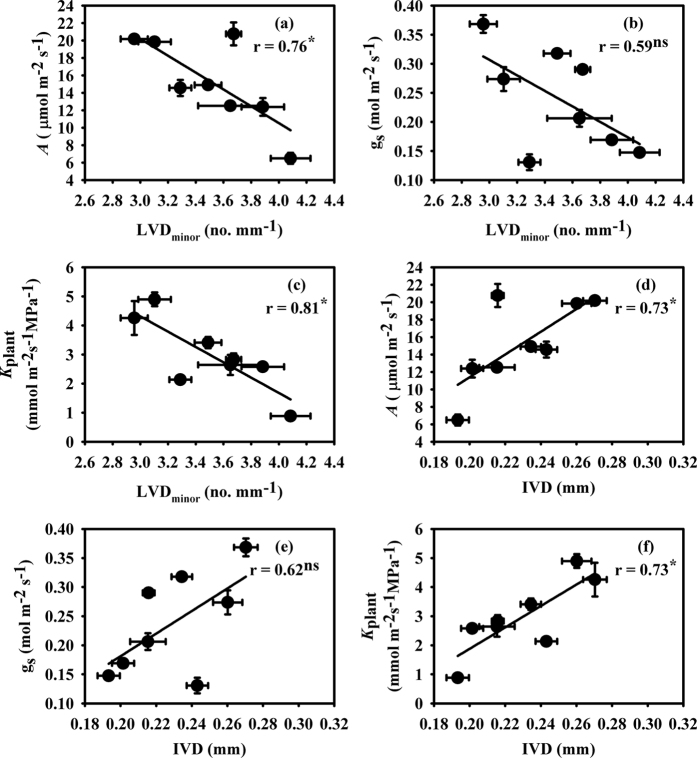
Relationships of photosynthesis (*A*) (**a**), stomatal conductance (g_s_) (**b**) and plant hydraulic conductance (*K*_plant_) (**c**) with minor leaf vein density (LVD_minor_) and inter vein distance (IVD) (**d**–**f**). The data are presented as the mean values of 3 replicates. ns, not significant; *P < 0.05.

Leaf size was decreased under all PEG-IWADS conditions in Hanyou-3, but in IR-64, it was only significantly decreased under 15% PEG-IWDS (Table 2). Compared with IR-64, a more severe decrease in leaf size was observed in Hanyou-3 under all PEG-IWDS conditions. Leaf size showed positive correlation with the major, and minor vein thickness as well as with inter-vein distances (IVD), while it showed negative correlations with LVD and LVD_minor_ (data not shown). LVD and LVD_minor_ showed non-significant increases in both varieties under all PEG-IWADS conditions. Interestingly, IR-64 had a higher leaf vein density than Hanyou-3 under all treatment conditions. On the other hand, IVD decreased non-significantly under all treatment conditions in both varieties, and Hanyou-3 had a higher IVD than IR-64. LVD had a negative correlation with *A* and *K*_plant_, but a non-significant relationship with g_s_ (Fig. 2). Similarly, LVD_minor_ had negative correlations with *A* and *K*_plant_ (Fig. 3a,c), but g_s_ was not significantly related to LVD_minor_ (Fig. 3b). IVD was positively correlated with *A* and *K*_plant_ (Fig. 3d,f) and was not related to g_s_ (Fig. 3e).

**Figure 4:**
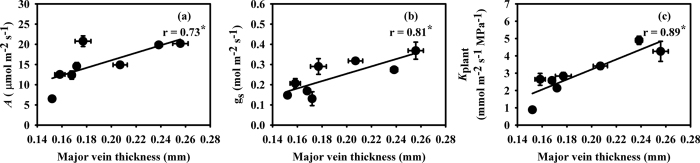
Relationships of photosynthesis (*A*) (**a**), stomatal conductance (g_s_) (**b**) and plant hydraulic conductance (*K*_plant_) (**c**) with leaf major vein thickness. The data are presented as the mean values of 4 replicates. *P < 0.05.

Major vein thickness decreased significantly in Hanyou-3 under 10 and 15% PEG-IWDS while a non-significant decrease was observed in IR-64 under all PEG-IWDS conditions (Table 3). Minor vein thickness decreased significantly in Hanyou-3 under 5% PEG-IWDS but non-significantly decreased under 10 and 15% PEG-IWDS. Moreover, the decrease in minor vein thickness was non-significant in IR-64 under all PEG-IWDS conditions. Major vein thickness showed a positive correlation with *A*, g_s_ and *K*_plant_ (Fig. 4). However, leaf minor vein thickness did not show any significant relationship with gas exchange or *K*_plant_ (data not shown).

## Discussion

Stomatal closure in response to water deficit stress will limit photosynthesis by restricting CO_2_ entry from the ambient environment into the intercellular air spaces of mesophyll cells^45^,^46^,^47^,^48^. Moreover, decreased g_m_ and impaired biochemical processes are non-stomatal limitations to photosynthesis that occur under severe or long-term water deficit conditions^49^,^50^,^51^. It is therefore logical that photosynthesis exhibited positive correlations with g_s_ or/and g_m_ in previous studies^52^,^53^,^54^,^55^. In the current study, *A* was also positively correlated with g_s_ (Fig. 1a). Chaves *et al.*^7^ reported that increased VPD and reduced turgor potential are major causes of stomatal closure under water-limited conditions. However, it is the boundary layers (leaf and canopy), as well as the driving force (VPD), that determine *E*, while *K*_plant_ determines the water potential at that *E*^9^,^10^. Thus, a high *K*_plant_ can maintain a high g_s_ and the consequent *A* without leading to desiccation of the plant leaves^14^,^56^,^57^,^58^,^59^. Linear correlations between *K*_plant_ and *E* or g_s_ were previously found in a number of higher plant species^15^,^16^,^17^,^18^. *K*_leaf_ is a major component of *K*_plant_, the positive correlation between *K*_plant_ and gas exchange may be related to *K*_leaf_. In the present study, the positive correlations between g_s_, *A* and *K*_plant_ suggest that *K*_plant_ is one of the key regulators of photosynthesis (Fig. 1).

The environmental signals present before and during leaf development determines the vein traits, like other leaf traits including leaf size and stomatal density^60^,^61^. Plasticity in vein traits was observed within the canopy and across environments for a given plant species. In this study, plasticity in leaf size, LVD, IVD and vein thickness were also observed under different PEG-IWDS conditions. Sack *et al.*^62^ suggested that LVD has a key influence on hydraulic conductance, g_s_ and *A*, and LVD is positively correlated with *A*. In the present study, *A* was negatively correlated with LVD and LVD_minor_ (Figs 2a and 3a) and positively correlated with IVD (Fig. 3d). This negative relationship between *A* and LVD is in accordance with negative relationships reported in angiosperms^63^,^64^,^65^,^66^,^67^, but different from the study by Xiong *et al.*^68^, who did not observe any relationship between g_s_, *A* and LVD during studies of the *Oryza* genus under well-watered condition.

Rice leaves are small and have more highly lobed mesophyll cells than C_4_ crop species^69^. They also have a lower LVD than C_4_ crops due to the higher number of mesophylls between veins. C_4_ plants, such as *Setaria viridis* and sorghum, have seven veins per millimeter, but rice has fewer than six veins per millimeter^70^. Although rice (C_3_) and maize (C_4_) both belong to the tropical-warm temperate grass family, rice has higher rates of photorespiration. This higher rate of photorespiration decreases the photosynthetic capacity by 30–35% at 30–35 °C ambient temperature^71^, and drought conditions make this more severe, so rice does not attain the full potential photosynthesis like C_4_ plants.

*K*_plant_ was negatively correlated with LVD (Fig. 2c), while it had a positive correlation with IVD (Fig. 3f). Mesophyll cells are more numerous in C_3_ than C_4_ plants, which increases IVD in C_3_ plants^70^,^72^ and reduces their *K*_leaf_^23^,^43^. Smillie *et al.*^73^ reported that IVD of rice plants is more dependent on cell size than cell number, which suggests that the lower IVD under water deficit conditions is mostly a result of more tightly packed, small mesophyll cells. The tightly packed mesophyll cells in smaller leaves under water deficit (Table 2) would produce more resistance in the apoplastic pathway for water transport in leaves, which would decrease *K*_leaf_ and the subsequent *K*_plant_.

Vein size also decreases under drought stress in addition to leaf vein differentiation. Martre and Durand^74^ reported that the vascular tissue is composed of xylem and phloem cells, and it carries out the transportation of different compounds. The flow rate of this transportation is determined by the size of the xylem and phloem cells. Decreases in the diameters of the xylem and phloem vessels were observed in *Ctenanthe setosa, Vigna unguiculata* and *Triticum aestivum* under water deficient conditions^75^,^76^,^77^, likely because the thin xylem vessels provide protection from cavitation under water-limited conditions^78^. In the present study, the major and minor vein thicknesses were also decreased under PEG-IWDS. The Hanyou-3 and IR-64 varieties showed more severe decreases in the major vein thickness (32.8% and 14.1%) than in the minor vein thickness (15.9% and 1.3%) under 15% PEG-IWDS (Table 3). The major, minor (longitudinal) and transverse veins have different sizes and functions^29^,^30^,^31^,^33^. The major leaf veins are the main supply lines for receiving water directly from the roots via the stem and leaf sheath, as they run from the leaf blade into the sheath while minor veins terminate at the junction of the leaf blade and sheath^34^,^35^,^36^,^37^. Water absorbed by the roots rises through the major veins from the leaf base to the leaf tips. After exiting the major veins, water reaches the minor veins via transverse veins and is finally distributed to mesophyll cells or is used for transpiration via stomata^31^,^32^,^33^. Ocheltree *et al.*^79^ suggested that g_s_ is strongly correlated with extra vascular resistance (outside large veins) under normal water regimes, while large vein resistance has a strong correlation with g_s_ under drought conditions. The current study suggests that the decreased major vein thickness that occurred under PEG-IWDS would increase the major vein resistance and restrict water uptake from the roots to leaves, and hence decreased *K*_plant_ and subsequently g_s_ and *A*.

Based on the present findings, we conclude that PEG-IWDS deceases *K*_plant_, photosynthesis, leaf vein thickness and IVD, while it increases LVD and LVD_minor_. LVD is negatively correlated with *K*_plant_ and photosynthesis, while major vein thickness is positively correlated with *K*_plant_, g_s_ and *A* under PEG-IWDS condition in rice crops.

## Materials and Methods

### Plant materials

Two rice cultivars, Hanyou-3 and IR-64, were selected because they had different drought tolerances with regard to photosynthesis in previous study. Hanyou-3 is considered drought-tolerant, while IR-64 is considered drought-sensitive. Seeds were surface-sterilized for 90 minutes using 10% H_2_O_2_, then washed with tap water to remove any residual H_2_O_2_. The seeds were germinated on moist filter paper until the radical emerged in the laboratory, then they were transferred to a seedling tray with tap water under natural environmental conditions. Seedlings were supplied with 1/8^th^-strength Hoagland solution on the fifth day of germination to avoid nutrient deficiency. Seedlings were transplanted after fifteen days of germination. Each bucket contained 10.5 L Hoagland solution. Seedlings were transplanted using a split block design such that each bucket had four seedlings of each variety. This experiment had six replicates and four treatments: the well-watered condition (WWC) and 5%, 10% and 15% (w/v) PEG-IWDS. Treatments were applied when seedlings reached 40 days of age. The composition of the full strength nutrient solution was as follows: macronutrients (mg l^−1^): 40 N as (NH_4_)_2_SO_4_ and Ca(NO_3_)_2_, 10 P as KH_2_PO_4_, 40 K as K_2_SO_4_ and KH_2_PO_4_, and 40 Mg as MgSO_4_; micronutrients (mg l^−1^): 2.0 Fe as Fe-EDTA, 0.5 Mn as MnCl_2_∙4H_2_O, 0.05 Mo as (NH_4_)_6_Mo_7_O_24_∙4H_2_O, 0.2 B as H_3_BO_3_, 0.01 Zn as ZnSO_4_∙7H_2_O, 0.01 Cu as CuSO_4_∙5H_2_O, 2.8 Si as Na_2_SiO_3_∙9H_2_O. Dicyandiamide was added to the nutrient solution as a nitrification inhibitor. Solutions were changed every fifth day, and the pH was maintained at 5.50 ± 0.05 every day by adding 0.1 molL^−1^HCl or NaOH. The experiment was conducted under natural environmental conditions in Huazhong Agricultural University (114.37E, 30.48N) Wuhan, Hubei, China.

### Gas exchange measurements

The gas exchanges were measured inside a growth chamber to avoid the fluctuations of the outdoor environment. The photosynthetic photon flux density (PPFD) was controlled to 1,000 μmol m^−2 ^s^−1^ using T5 fluorescent lamps and halogen incandescent lamps fixed on a down and upward moving panel. There were three fans built in the roof of the growth chamber to avoid over-heating of the growth chamber, and the air temperature was set to 30/25 °C day/night with 11 h photoperiod. The relative humidity in the growth chamber was controlled at 65%..

### Leaf area measurement

Three newly expanded leaves for each variety and replicate were detached, followed by leaf area measurement using a leaf area meter (Li-Cor 3000 C, Li-Cor, NE, USA).

### Leaf vein density measurement

Rice leaf veins were divided into three categories based on their size (i.e., midrib, major and minor veins) to calculate the leaf vein density^73^. One centimeter leaf sections were excised with a razor blade from the middle portion of newly-developed leaves after measuring the leaf width. These sections were immediately immersed in tap water and carried to a laboratory to observe all visible longitudinal leaf vein numbers. In the laboratory, all visible leaf veins (sum of the midrib, major and minor leaf veins) were counted under 40x magnification using a light microscope (SA3300, Beijing Tech Instrument Co., Ltd, Beijing, China). IVD was calculated by dividing the leaf width with the respective total longitudinal leaf vein numbers. LVD was calculated as total vein length per leaf area, and LVD_minor_ was calculated as the total minor vein length per leaf area.

### Measurement of plant hydraulic conductance

During the gas exchange measurements, newly and fully developed leaves were used to measure the day time leaf water potential using a WP4C Dewpoint Potential Meter (Decagon, Pullman, WA, USA). *K*_plant_ was calculated following the formula described by Brodribb and Holbrook^81^:


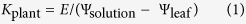


where Ψ_solution_ was 0 for WWC, and was −0.05, −0.18 and −0.38 MPa, respectively, for the 5%, 10% and 15% PEG-IWDS.

### Leaf vein thickness measurement

Minor vein thickness was measured for each side of the leaf (avoiding midribs) using a leaf thickness measuring instrument (YI-20030A, China Jiliang University), while major vein thickness was measured using a DTG03 digital thickness gauge (Digital Micrometers Ltd, Sheffield, UK).

### Statistical analysis

One and two-way analyses of variance (ANOVA) were applied to assess the differences between treatments with Statistics 8.1 analytical software. Linear regression and correlation analysis were performed to test the possible correlations between the studied parameters using Sigma Plot 12 (SPSS Inc., Chicago, IL, USA).

## Additional Information

**How to cite this article**: Tabassum, M. A. *et al.* Influence of leaf vein density and thickness on hydraulic conductance and photosynthesis in rice (*Oryza sativa* L.) during water stress. *Sci. Rep.* **6**, 36894; doi: 10.1038/srep36894 (2016).

**Publisher’s note:** Springer Nature remains neutral with regard to jurisdictional claims in published maps and institutional affiliations.

## Figures and Tables

**Table 1:** Effects of PEG-induced water deficit stress on photosynthesis (*A*), stomatal conductance (g_s_), intercellular CO_2_ concentration (C_i_), transpiration rate (*E*) and leaf water potential (Ψ_leaf_) of newly-developed leaves of two rice varieties at the vegetative stage.

Varieties	Treatment	*A* (μmol m^−2 ^s^−1^)	g_s_ (mol m^−2 ^s^−1^)	C_i_ (μmol mol^−1^)	*E* (mmol m^−2 ^s^−1^)	Ψ_leaf_ (MPa)	*K*_plant_ (mmol m^−2 ^s^−1^ MPa^−1^)
Hanyou-3	WWC	20.2 ± 0.3a	0.37 ± 0.04a	236 ± 4	6.29 ± 0.86a	−1.48 ± 0.03bc	4.26 ± 0.58a
	PEG-IWDS5%	19.9 ± 0.3a	0.27 ± 0.01ab	258 ± 3	6.45 ± 0.31a	−1.37 ± 0.02a	4.90 ± 0.24a
	PEG-IWDS10%	14.9 ± 0.4b	0.32 ± 0.00ab	312 ± 4	4.07 ± 0.23ab	−1.37 ± 0.03ab	3.41 ± 0.19ab
	PEG-IWDS15%	14.6 ± 0.9b	0.13 ± 0.03b	277 ± 27	2.82 ± 0.04b	−1.70 ± 0.02c	2.13 ± 0.03b
IR-64	WWC	20.8 ± 1.3a	0.29 ± 0.04a	258 ± 10	4.44 ± 0.32a	−1.57 ± 0.04ab	2.83 ± 0.21a
	PEG-IWDS5%	12.5 ± 0.4b	0.21 ± 0.02ab	263 ± 4	3.77 ± 0.49a	−1.48 ± 0.02a	2.64 ± 0.35a
	PEG-IWDS10%	12.4 ± 1.0b	0.17 ± 0.02b	260 ± 6	3.27 ± 0.46a	−1.44 ± 0.06ab	2.98 ± 0.15a
	PEG-IWDS15%	6.5 ± 0.6c	0.15 ± 0.01b	314 ± 3	1.30 ± 0.08b	−1.85 ± 0.02b	0.88 ± 0.05b
ANOVA							
Treatment (T)		**	**	ns	**	**	*
Variety (V)		ns	ns	ns	ns	ns	*
T × V		**	ns	ns	ns	ns	ns

Water deficit stress was simulated by adding 5, 10 or 15% (W/V) PEG6000 to the nutrient solution.

WWC, well-watered condition; PEG-IWDS, PEG-induced water deficit stress. The data are presented as the means ± SE with 3 replicates. ns, not significant; *P < 0.05, **P < 0.01, ***P < 0.001. The data followed by the different letters of each variety within a single column are significant at *P* < 0.05 level.

**Table 2:** Effects of PEG-induced water deficit stress on the single leaf area, leaf vein density (LVD), minor leaf vein density (LVD_minor_), and inter-vein distance (IVD) of newly developed leaves of two rice varieties at the vegetative stage.

Varieties	Treatment	Single leaf area (cm^2^)	LVD (no. mm^−1^)	LVD_minor_ (no. mm)	IVD (mm)
Hanyou-3	WWC	72.3 ± 1.1a	3.71 ± 0.09a	2.96 ± 0.10a	0.270 ± 0.007a
	PEG-IWDS5%	53.3 ± 2.8b	3.87 ± 0.13a	3.10 ± 0.12a	0.260 ± 0.008a
	PEG-IWDS10%	52.5 ± 1.6bc	4.28 ± 0.10a	3.49 ± 0.10a	0.234 ± 0.006a
	PEG-IWDS15%	40.0 ± 1.4c	4.13 ± 0.11a	3.29 ± 0.08a	0.243 ± 0.006a
IR-64	WWC	25.9 ± 0.4a	4.64 ± 0.07a	3.67 ± 0.05a	0.216 ± 0.003a
	PEG-IWDS5%	21.7 ± 0.9ab	4.71 ± 0.23a	3.65 ± 0.23a	0.215 ± ± 0.010a
	PEG-IWDS10%	22.7 ± 1.2ab	4.99 ± 0.16a	3.88 ± 0.15a	0.201 ± 0.006a
	PEG-IWDS15%	19.4 ± 0.2b	5.21 ± 0.17a	4.08 ± 0.14a	0.193 ± 0.006a
ANOVA					
Treatment (T)		***	ns	ns	ns
Variety (V)		***	**	*	*
T × V		*	ns	ns	ns

Water deficit stress was simulated by adding 5, 10 or 15% (W/V) PEG6000 to the nutrient solution.

WWC, well-watered condition; PEG-IWDS, PEG-induced water deficit stress. The data are presented as the means ± SE with 3 replicates. ns, not significant; *P < 0.05, **P < 0.01, ***P < 0.001. The data followed by the different letters of each variety within a single column are significant at *P* < 0.05 level.

**Table 3:** Effects of PEG-induced water deficit stress on the leaf major and minor vein thickness of newly developed leaves of two rice varieties at the vegetative stage.

Varieties	Treatment	Major vein thickness (mm)	Minor vein thickness (mm)
Hanyou-3	WWC	0.256 ± 0.006a	0.120 ± 0.007a
	PEG-IWDS5%	0.238 ± 0.003a	0.093 ± 0.002b
	PEG-IWDS10%	0.207 ± 0.006b	0.100 ± 0.001ab
	PEG-IWDS15%	0.172 ± 0.002c	0.101 ± 0.001ab
IR-64	WWC	0.177 ± 0.006a	0.098 ± 0.002a
	PEG-IWDS5%	0.158 ± 0.004a	0.092 ± 0.001a
	PEG-IWDS10%	0.168 ± 0.003a	0.092 ± 0.001a
	PEG-IWDS15%	0.152 ± 0.002a	0.096 ± 0.001a
ANOVA			
Treatment (T)		***	ns
Varieties (V)		***	ns
T × V		*	ns

Water deficit stress was simulated by adding 5, 10 or15% (W/V) PEG6000 to the nutrient solution.

WWC, well-watered condition; PEG-IWDS, PEG-induced water deficit stress. The data are presented as the means ± SE with 3 replicates. ns, not significant; *P < 0.05, **P < 0.01, ***P < 0.001. The data followed by the different letters of each variety within a single column are significant at *P* < 0.05 level.
